# Identification and characterization of novel amphioxus microRNAs by Solexa sequencing

**DOI:** 10.1186/gb-2009-10-7-r78

**Published:** 2009-07-17

**Authors:** Xi Chen, Qibin Li, Jin Wang, Xing Guo, Xiangrui Jiang, Zhiji Ren, Chunyue Weng, Guoxun Sun, Xiuqiang Wang, Yaping Liu, Lijia Ma, Jun-Yuan Chen, Jun Wang, Ke Zen, Junfeng Zhang, Chen-Yu Zhang

**Affiliations:** 1 Jiangsu Diabetes Center, State Key Laboratory of Pharmaceutical Biotechnology, School of Life Sciences, Nanjing University, Hankou Road, Nanjing, Jiangsu 210093, PR China; 2 Beijing Institute of Genomics, Chinese Academy of Sciences, Beitucheng West Road, Chaoyang District, Beijing 100029, PR China; 3 Beijing Genomics Institute, Beishan Road, Yantian District, Shenzhen 518083, PR China; 4 Graduate University of Chinese Academy of Sciences, Yuquan Road, Shijingshan District, Beijing 100049, PR China; 5 Nanjing Institute of Palaeontology and Geology, East Beijing Road, Nanjing, Jiangsu 210008, PR China

## Abstract

**Background:**

microRNAs (miRNAs) are endogenous small non-coding RNAs that regulate gene expression at the post-transcriptional level. While the number of known human and murine miRNAs is continuously increasing, information regarding miRNAs from other species such as amphioxus remains limited.

**Results:**

We combined Solexa sequencing with computational techniques to identify novel miRNAs in the amphioxus species *B. belcheri *(Gray). This approach allowed us to identify 113 amphioxus miRNA genes. Among them, 55 were conserved across species and encoded 45 non-redundant mature miRNAs, whereas 58 were amphioxus-specific and encoded 53 mature miRNAs. Validation of our results with microarray and stem-loop quantitative RT-PCR revealed that Solexa sequencing is a powerful tool for miRNA discovery. Analyzing the evolutionary history of amphioxus miRNAs, we found that amphioxus possesses many miRNAs unique to chordates and vertebrates, and these may thus represent key steps in the evolutionary progression from cephalochordates to vertebrates. We also found that amphioxus is more similar to vertebrates than are tunicates with respect to their miRNA phylogenetic histories.

**Conclusions:**

Taken together, our results indicate that Solexa sequencing allows the successful discovery of novel miRNAs from amphioxus with high accuracy and efficiency. More importantly, our study provides an opportunity to decipher how the elaboration of the miRNA repertoire that occurred during chordate evolution contributed to the evolution of the vertebrate body plan.

## Abstract 2

An analysis of amphioxus miRNAs suggests an expansion of miRNAs played a key role in the evolution of chordates to vertebrates

## Background

When the class of RNA regulatory genes known as microRNAs (miRNAs) was discovered it introduced a whole new layer of gene regulation in eukaryotes [1]. Since the discovery of the first miRNA (*lin-4*) in *Caenorhabditis elegans*, thousands of miRNAs have been identified experimentally or computationally from a variety of species [1]. miRNAs are currently estimated to comprise 1 to 5% of animal genes and collectively regulate up to 30% of genes, making them one of the most abundant classes of regulators [2]. However, while the importance of miRNAs in animal ontogeny has been rapidly elucidated, their role in phylogeny currently remains largely unknown. Recent studies have provided important clues indicating that these approximately 22-nucleotide non-coding RNAs might have been a causative factor in increasing organismal complexity through their action in regulating gene expression [3-6]. Indeed, vertebrates possess many more miRNAs than any invertebrate sampled to date, and the emergence of vertebrates is characterized by an unprecedented increase in the rate of miRNA family innovation [4-6]. However, how this increase in the miRNA repertoire relates to the emergence of the complex vertebrate body plan is currently unclear because groups from which we might gain insight into this (such as amphioxus) have not been thoroughly studied yet.

**Figure 1:**
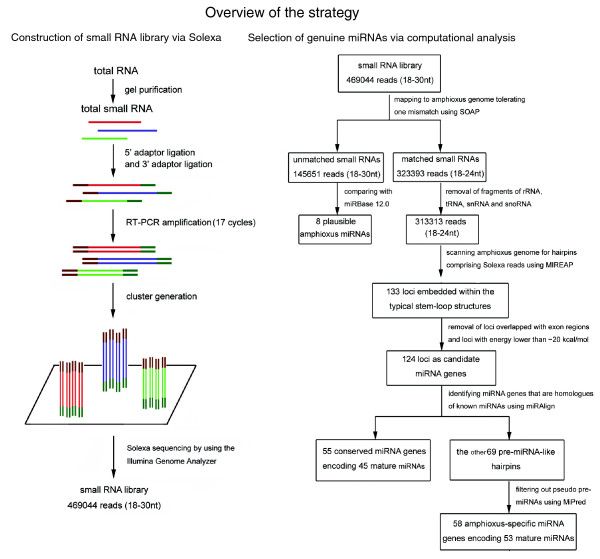
Step-by-step schematic description of the strategy for amphioxus miRNA discovery and validation. nt = nucleotide; snRNA = small nuclear RNA; small nucleolar RNA.

As the living invertebrate relative of the vertebrates, amphioxus affords the best available glimpse of a proximate invertebrate ancestor of the vertebrates and is likely to exemplify many of the starting conditions at the dawn of vertebrate evolution [7,8]. The completion of the amphioxus genome project provides a tremendous opportunity for identifying miRNAs in this organism [9]. According to the rules proposed by Ambros *et al*. [10] and Berezikov *et al*. [11], a genuine miRNA should fulfill two basal requirements for miRNA annotation: its expression should be confirmed experimentally (the expression criterion) and the putative miRNA should be embedded within a canonical stem-loop hairpin precursor (the structural criterion). Furthermore, an optional but commonly used criterion is that the mature miRNA sequence and the predicted hairpin structure should be conserved in different species. Non-conserved miRNAs require more careful examination. In this work, we have proposed an integrative strategy combining an experimental screen with bioinformatic analysis to identify miRNAs fulfilling all these requirements (Figure 1). Our strategy has four steps: investigating all small RNAs expressed in the amphioxus *Branchiostoma belcheri *(Gray) via Solexa, a massively parallel sequencing technology [12]; computationally scanning the amphioxus genome (*Branchiostoma floridae *v2.0) for candidate hairpin miRNA genes corresponding to Solexa reads using MIREAP; identifying conserved miRNA genes using miRAlign [13]; and distinguishing functional non-conserved miRNA precursors (pre-miRNAs) from dysfunctional pseudo-hairpins using MiPred [14]. Our approach allows the simultaneous sequencing of up to 400,000 small RNA reads in a lane, and enables the identification of both conserved miRNAs and completely new miRNAs for which no close homologs are known. Using this method, we obtained experimental evidence for 113 miRNA genes in the amphioxus *B. belcheri *(Gray), of which 55 are conserved and 58 are amphioxus-specific. The genomic organization and evolution history of these amphioxus miRNAs were also characterized.

## Results

### Construction of a small RNA library by Solexa sequencing

In order to identify the miRNAs in amphioxus, a small RNA library from adult amphioxus was sequenced using Solexa technology [12]. After removing the reads of low quality and masking adaptor sequences, a total of 469,044 reads of 18 to 30 nucleotides in length were obtained. Solexa raw data are available at Gene Expression Omnibus [GEO:GSE16859]. Intriguingly, the length distribution peaked at 22 nucleotides and almost half of these clean reads (45.11%) were 22 nucleotides in length, consistent with the common size of miRNAs. This result implies an enrichment of miRNA in the small RNA library of amphioxus. Next, all Solexa reads were aligned against the amphioxus genome (*Branchiostoma floridae *v2.0) using SOAP (Short Oligonucleotide Alignment Program) [15] with a tolerance of one mismatch. The results indicated that 257,746 reads were perfectly matched to the amphioxus genome and 65,647 reads differed from the amphioxus genome by one nucleotide (323,393 reads in total).

Subsequently, the amphioxus small RNAs were classified into different categories according to their biogenesis and annotation (Table S1 in Additional data file 1). Among the 323,393 genome-matched reads, 3,420, 6,438, 210, and 12 were fragments of rRNA, tRNA, small nuclear RNA (snRNA), and small nucleolar RNA (snoRNA), respectively. These RNAs were abandoned and the remaining 313,313 small RNAs were retained for further analysis.

### Selection of genuine miRNAs by computational analysis

One of the important features that distinguish miRNAs from other endogenous small RNAs is the ability of the pre-miRNA sequence to adopt a canonical stem-loop hairpin structure [10,11]. To determine whether these small RNA sequences from amphioxus were genuine miRNAs, we scanned the amphioxus genome (*Branchiostoma floridae *v2.0) for hairpin structures comprising the candidate miRNAs using our in-house software MIREAP, which was specially designed to identify genuine miRNAs from deeply sequenced small RNA libraries. In total, our *in silico *analysis generated 133 loci embedded within typical stem-loop structures (Table S2 in Additional data file 1). After the removal of five loci that overlapped with protein-coding gene exons and four loci with free energy lower than -20 kcal/mol (see the criteria listed in Materials and methods), the remaining 124 loci were considered candidate miRNA genes (Table S3 in Additional data file 1).

Subsequently, we used miRAlign to identify miRNA genes of amphioxus that are paralogs or orthologs to known miRNAs. miRAlign is a computational approach that detects new miRNAs based on both sequence and structure alignment, and it has better performance than other reported homolog searching methods [13]. We applied this method to the 124 candidate miRNA genes and detected 55 conserved miRNA genes (Table 1; Table S5 in Additional data file 1; Additional data file 2). Among 55 miRNA genes, 36 are present as a single copy in the amphioxus genome, while 9 have multiple copies distributed on the same or separate chromosomes that produce identical mature miRNAs (Table 1). In total, 45 non-redundant mature miRNAs were encoded by these conserved miRNA genes (Table 1; Table S4 in Additional data file 1). Simultaneously, 27 miRNA*s were detected (Table 1; Table S4 in Additional data file 1). Since the mature sequences for miRNAs and miRNA*s are located at two opposite arms of the hairpin [1], the detection of miRNA* sequences supports the release of miRNA:miRNA* duplexes from the predicted stem-loop structure. Among the 45 conserved miRNAs, 10 were identical with known miRNAs, 8 had one nucleotide mismatch, 16 had two nucleotide differences, 5 contained three mismatches, and 6 had 4 to 5 mismatches (Table S4 in Additional data file 1). All of these mismatches were located outside the 'seed' region (the core sequence that encompasses the first two to eight bases of the mature miRNA). In contrast to the amphioxus miRNAs, which showed high similarity to miRNAs from other organisms (mismatches ≤ 3), most amphioxus miRNA*s differed from the known miRNA* by three to five nucleotides (data not shown). This result suggests that miRNA*s are less conserved than miRNAs.

**Table 1:** Number of novel miRNAs sequenced from amphioxus by Solexa technology

	miRNA genes present as a single copy	miRNA genes present as two copies	miRNA genes present as three copies	Total miRNA genes	Mature miRNAs	Mature miRNA*s
Conserved miRNA genes	36	8	1	55	45	27
Amphioxus-specific miRNA genes	49	3	1	58	53	18
Sum				113	98	45

Obviously, methods that rely on phylogenetic conservation of the structure and sequence of a miRNA cannot predict non-conserved genes. However, a substantial number of species-specific miRNA genes have been found that escaped the detection of comparative genomics approaches [16]. On the other hand, although the hairpin structure is a necessary feature for the computational classification of genuine pre-miRNA, many random inverted repeats (termed pseudo-hairpins) in eukaryotic genomes can also fold into dysfunctional hairpins [14,17]. Thus, additional care should be taken to classify functional non-conserved miRNAs. To overcome this problem, several *ab initio *predictive approaches have been extensively developed for identifying pre-miRNAs without relying on phylogenetic conservation [14,17]. Here, we adopted an *ab initio *prediction method named MiPred to distinguish pre-miRNAs from other similar segments in the amphioxus genome [14]. Unlike comparative genomics approaches, MiPred relies solely on secondary structure to evaluate miRNA candidates and, therefore, can estimate species-specific miRNAs without knowing sequence homology [14,17]. Furthermore, it has been reported that MiPred performs as well or significantly better (in terms of sensitivity and specificity) than existing classifiers at distinguishing non-conserved functional pre-miRNAs from genomic pseudo-hairpins and non-pre-miRNAs (most classes of non-coding RNAs and mRNAs) [17]. Among the remaining 69 pre-miRNA-like hairpins, 11 were classified as pseudo-pre-miRNAs (Table S3 in Additional data file 1). Thus, the final collection of amphioxus-specific miRNA genes is composed of 58 loci (Table 1; Table S5 in Additional data file 1; Additional data file 3) that encode 53 non-redundant mature miRNAs (Table 1; Table S4 in Additional data file 1). Herein, we tentatively designate them bbe-miR-specific-1 (bbe-miR-s1), bbe-miR-s2, bbe-miR-s3, bbe-miR-s4, and so on. Among these amphioxus-specific miRNA genes, the miRNA* sequences of 18 genes were identified (Table 1; Table S4 in Additional data file 1), further supporting their existence as miRNAs in amphioxus.

**Figure 2:**
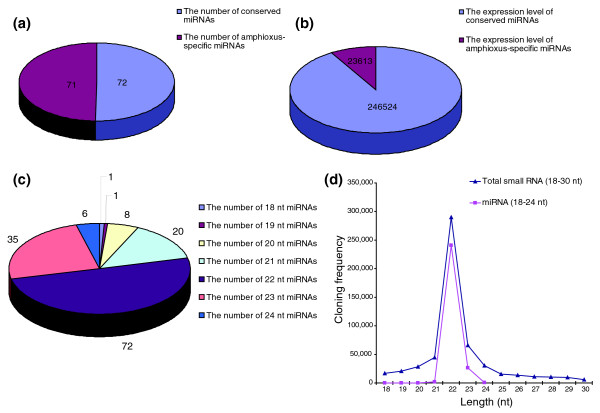
Characterization of amphioxus miRNAs. **(a, b) **Comparison of the number (a) and absolute sequencing frequency (b) of conserved miRNAs with those of amphioxus-specific miRNAs. **(c) **The composition of amphioxus miRNAs of various lengths (in nucleotides (nt)). **(d) **The size distribution of small amphioxus RNAs and miRNAs of various lengths sequenced by Solexa.

The sequencing frequency of the miRNAs generally reflected their relative abundance and was, therefore, used to establish miRNA expression profiles (Table S4 in Additional data file 1). Although the 98 miRNAs (45 conserved + 53 non-conserved) and 45 miRNA*s were sequenced at varying frequencies, some miRNAs dominated the miRNA library. The sequencing frequency of the four most abundantly expressed miRNAs (miR-22, miR-1, *let-7a *and miR-25) constituted 78.82% of the total miRNA sequencing reads, suggesting that they might be ubiquitously expressed in amphioxus. In contrast, the sequencing frequency of miR-129, miR-s53, miR-s26, miR-s31, miR-s46, and so on was extremely low in our library. It is possible that these miRNAs are expressed at very low levels, in limited cell types, and/or under limited circumstances. Most miRNA*s showed weak expression (sequencing frequency < 10) and their expression levels were much lower than their corresponding miRNAs, consistent with the idea that miRNA* strands are degraded rapidly during the biogenesis of mature miRNAs. Furthermore, although the number of amphioxus-specific miRNAs was nearly equal to that of the conserved miRNAs (Figure 2a), the absolute sequencing frequencies of the amphioxus-specific miRNAs was much lower (Figure 2b). The miRNA size distribution ranged from 18 to 24 nucleotides, with 22 nucleotides the most abundant both in number (50.70%) and sequencing frequency (89.23%) (Figure 2c, d). Analysis of the nucleotides at the ends of these miRNAs revealed that uridine (U) was the most common nucleotide both at the 5' end (54.87%) and the 3' end (64.60%).

In order to find more potential miRNAs in amphioxus, unmapped small RNAs were directly compared with the miRBase release 12.0 [18]. The search criteria were more rigorous, and required small RNAs to display a perfect or nearly perfect match (mismatch ≤ 1) to published miRNAs. Moreover, the mismatches were required to be outside the 'seed' region. Based on these principles, we identified eight candidate miRNAs (bbe-miR-21, bbe-miR-122, bbe-miR-192, and so on). We considered these small RNAs to be plausible amphioxus miRNAs (Table S6 in Additional data file 1). The reason that these sequencing reads were successfully matched to miRBase 12.0 but failed to match the *B. floridae *genome might be due to incomplete genome sequencing in *B. floridae *or to genomic divergence between *B. belcheri *(Gray) and *B. floridae*.

### Detection of amphioxus miRNA expression with stem-loop quantitative RT-PCR and microarray analysis

**Figure 3:**
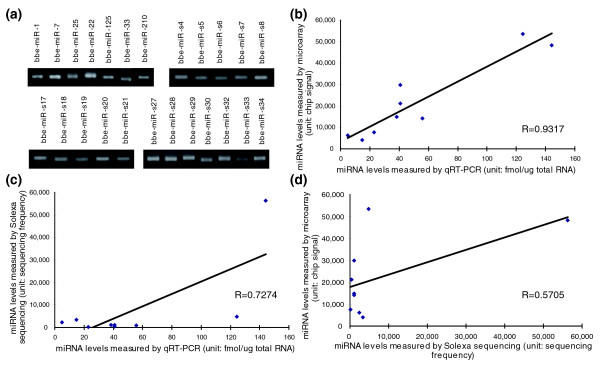
Confirmation of the accuracy of Solexa sequencing with qRT-PCR and microarray analysis. **(a) **The expression levels of the indicated miRNAs in amphioxus evaluated by semi-quantitative RT-PCR with 30 cycles. **(b-d) **Nine miRNAs (bbe-miR-1, bbe-miR-10a, bbe-miR-29b, bbe-miR-92a, bbe-miR-125, bbe-miR-184, bbe-miR-210, bbe-miR-216, and bbe-miR-217) were selected and their expression levels were measured by Solexa sequencing, stem-loop qRT-PCR and microarray analysis. The data obtained from each of these methods were then compared with the data obtained from each of the others and drawn as a Pearson correlation scatter plot.

To verify the existence of the newly identified amphioxus miRNAs, the same RNA preparation used in the Solexa sequencing was subjected to stem-loop quantitative RT-PCR (qRT-PCR) assay [19,20]. In total, all 45 conserved miRNAs and 50 out of 53 amphioxus-specific miRNAs (except bbe-miR-s1, bbe-miR-s31 and bbe-miR-s46) could be readily detected by stem-loop qRT-PCR. Figure 3a shows representative photographic images of the semi-quantitative RT-PCR. As shown in the figure, bbe-miR-1, bbe-let-7, bbe-miR-25, bbe-miR-22, and so on were clearly expressed in amphioxus. Therefore, these miRNAs are authentic miRNAs. In sum, these results suggest that Solexa sequencing is capable of successfully discovering novel miRNAs from this species with high accuracy and efficiency.

Moreover, we detected the expression of the newly identified miRNAs in amphioxus with microarray analysis [21]. Except for the amphioxus-specific miRNAs and five miRNAs (bbe-miR-71, bbe-miR-278, bbe-miR-252a, bbe-miR-252b, and bbe-miR-281) whose homologs were not contained in the available commercial microarray chips, 65% of the miRNAs (26 out of 40) could be detected by microarray analysis, and most undetected miRNAs had either low expression (sequencing frequency < 100) or a low affinity to chip probes (mismatches ≥ 3) (Table S7 in Additional data file 1). This result suggests that Solexa sequencing is a more specific tool for identifying mature miRNAs than miRNA microarray analysis. Another discordant observation is that seven miRNAs were detected in the microarray analysis but were undetected by the Solexa sequencing (Table S7 in Additional data file 1). These miRNAs need to be further validated in amphioxus. Table S8 in Additional data file 1 lists the raw miRNA microarray data.

Although the Solexa sequencing, stem-loop qRT-PCR assay and microarray analysis detected the same set of amphioxus miRNAs, the expression levels measured by these three platforms might be somewhat inconsistent for certain miRNAs. We chose nine miRNAs and compared their expression levels as measured by these three platforms. These miRNAs were selected because they could be detected by all three methods and because they had high affinity to the chip probes (mismatches ≤ 1). As shown in Figure 3b, expression levels measured by microarray and qRT-PCR assay were quite concordant, with a Pearson correlation coefficient (*R*) close to 1. In contrast, the levels measured by Solexa sequencing were inconsistent with those determined by microarray and qRT-PCR (Figure 3c, d). Thus, although Solexa sequencing is approved to be an accurate and efficient strategy for miRNA identification, it might be somewhat inferior to the more commonly used quantitative methodologies (qRT-PCR and microarray) for miRNA quantification. This discordance might be due to cloning bias or to sequencing bias inherent in the deep-sequencing approach. In addition, some miRNAs might be hard to sequence due to physical properties or post-transcriptional modifications such as methylation.

### miRNA gene clusters in the amphioxus genome

**Figure 4:**
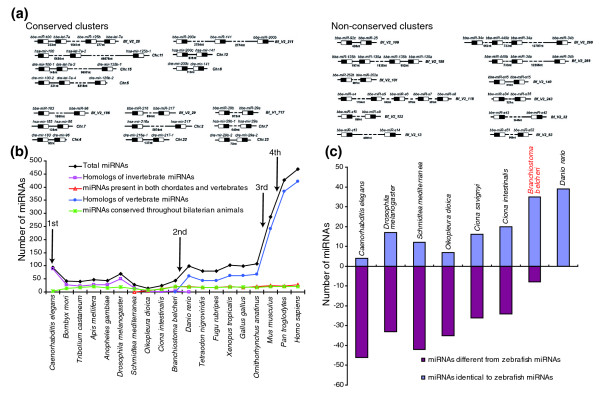
The phylogenetic histories of amphioxus miRNAs. **(a) **miRNA gene clusters in amphioxus. At a 3,000-nucleotide distance threshold, the amphioxus genome contains 17 compact clusters with 39 miRNAs. The precursor structure is indicated as a box, and the location of the miRNA within the precursor is shown in black. Some of these clusters in amphioxus are also conserved in zebrafish and humans. **(b) **The evolutionary histories of miRNAs and their relationship to the milestones of macroevolution. We integrated amphioxus miRNAs into the currently known miRNAs (miRBase release 12.0) and performed a comprehensive screening of their phylogenetic histories across animals. Each miRNA was classified into one of four groups: miRNAs conserved throughout bilaterian animals; homologs of invertebrate miRNAs; miRNAs present in both chordates and vertebrates; and homologs of vertebrate miRNAs. Note that our approach ignored species-specific miRNAs, since these miRNAs do not offer any information about miRNA evolution. **(c) **Comparison of the miRNA repertoires of amphioxus and tunicates. By using zebrafish as a reference, we compared the miRNA repertoires of nematodes, fruit flies, tunicates, and amphioxus. miRNAs with a zebrafish homolog were recorded as +1; miRNAs not found in zebrafish were recorded as -1.

miRNAs are often present in the genome as clusters where multiple miRNAs are aligned in the same orientation and transcribed as a polycistronic structure, allowing them to function synchronously and cooperatively [1]. Altuvia *et al*. [22] demonstrated that 42% of known human miRNA genes are arranged in clusters in the genome using a 3 kb threshold between two miRNA genes. We followed the strategies proposed by Altuvia *et al*. and defined 3,000 nucleotides as the maximal distance for two miRNA genes to be considered as clustered. By this definition, we identified 45 miRNA genes organized into 17 compact clusters, including 11 pairs, two triplets, three tetrads and one group of five (Figure 4a). Some of the amphioxus miRNA clusters are conserved within vertebrate species, implying an ancient origin conserved throughout the course of evolution. For example, the miR-183/miR-96 cluster in amphioxus was also found in humans and zebrafish (Figure 4a). In contrast, some clusters, such as the miR-s4/miR-s5/miR-s6/miR-s7/miR-s8 cluster, seem to be an amphioxus innovation (Figure 4a).

### Phylogenic history of amphioxus miRNAs

Previous studies have suggested that miRNA innovation is an ongoing process [3-6]. The most crucial morphological innovations during evolution are closely linked to the specific expression of a unique set of miRNA genes [3-6]. Herein, we extended the earlier studies by integrating amphioxus miRNAs into the currently known miRNAs (miRBase release 12.0) and performed a comprehensive screening of their phylogenetic histories across bilaterian animals. Based on the available nematode, fruitfly, zebrafish, frog, chicken, mouse, rat and human miRNA information [18], 45 conserved amphioxus miRNAs could be classified into three distinct groups: 23 miRNAs (*let-7a*, miR-1, miR-7, miR-9, and so on) were conserved throughout the Bilateria; 5 miRNAs (miR-252a, miR-252b, miR-278, miR-281 and miR-71) were homologous to invertebrate miRNAs; and 17 miRNAs (miR-141, miR-200a, miR-200b, miR-183, miR-216, miR-217, miR-25, miR-22, miR-96, and so on) were present both in chordates and vertebrates (Table S9 in Additional data file 1). The miRNAs present in both chordates and vertebrates but not in previous protostomes represent cephalochordate lineage innovation, and this may advance our understanding of the homology between the body plans of amphioxus and vertebrates.

In agreement with previous studies [3-6], we also observed an acquisition of miRNA genes across the evolutionary step from lower metazoans to higher vertebrates. Four major episodes of miRNA innovation, correlated with significant body plan changes among animals, have been identified since the advent of Bilateria (Figure 4b; total miRNAs). The first wave of miRNA innovation maps to the origin of bilaterian miRNAs. The second wave maps to the branch leading to the vertebrates. The third wave of miRNA expansion corresponds to the advent of eutherian mammals. The fourth wave of miRNA outburst coincides with the advent of primates. This observation strengthens the view that miRNAs have an important role in shaping animal phenotypic diversity and complexity. However, the expansion of the miRNA repertoire in the cephalochordate lineage does not correspond to the outburst of miRNA innovation. Approximately 20 miRNAs are shared throughout the Bilateria, and all of these exist in amphioxus (Figure 4b, miRNAs conserved throughout the Bilateria). These miRNAs are phylogenetically conserved despite several hundred million years of divergent evolution, suggesting ancient roles for them in activating the terminal differentiation of organs, tissues and specific cell types common to metazoans. Protostomes and chordates appear to have miRNAs that are specific to each clade as most invertebrate miRNAs have been lost in the chordate lineage (Figure 4b, homologs of invertebrate miRNAs), and many novel miRNAs present in both chordates and vertebrates have been fixed in the chordate genome and perpetuated under intense purifying selection over evolutionary time (Figure 4b, miRNAs present in both chordates and vertebrates). This observation suggests that chordates have abandoned most ancestral characters and are more vertebrate-like than any other invertebrate. Since many vertebrate miRNAs have homologs in amphioxus, these miRNAs must, therefore, have been present in the last common ancestor of vertebrates. Thus, the profound reorganization of the miRNA repertoires (the continuous expansion of the miRNA inventory and the loss of ancient miRNAs) in amphioxus highlights the importance of amphioxus as a model for understanding the transition from invertebrates to vertebrates.

### Comparison of the miRNA repertoires of cephalochordates and tunicates

miRNA can also be employed as a valuable factor to resolve outstanding evolutionary questions. For instance, a fundamental evolutionary question is whether cephalochordates or tunicates are the closest living invertebrate relative of the vertebrates [23]. Living invertebrate chordates comprise the urochordate tunicates (the most familiar of which are the ascidians) and the cephalochordate amphioxus. Traditionally, cephalochordates are considered to be the closest living relatives of vertebrates, with tunicates representing the earliest chordate lineage [7,8]. However, recent phylogenetic analyses with large concatenated gene sets suggest that the evolutionary positions of tunicates and cephalochordates should be reversed [24]. In order to solve this puzzle, we reconstructed the evolutionary histories of tunicates and cephalochordates according to their miRNA histories.

If tunicates are more vertebrate-like, then they should possess a subset of miRNAs conserved across chordates and vertebrates, but few invertebrate-specific miRNAs. However, by tracing the phylogenetic histories of miRNAs in *Oikopleura dioica*,*Ciona intestinalis*, and *B. belcheri *(Gray), we found that several phylogenetically conserved miRNAs were either lost or no longer recognizable in *Oikopleura dioica *(for example, miR-33, miR-34, miR-125, miR-133, miR-184, and miR-210), and we did not detect any miRNAs present in both chordates and vertebrates. Likewise, some phylogenetically conserved miRNAs were also lost in *C. intestinalis *(for example, miR-1, miR-9 and miR-10). In contrast, many phylogenetically conserved miRNAs, as well as miRNAs present in both chordates and vertebrates (for example, miR-216, miR-217, miR-22, miR-25, and miR-96), could be reliably traced back to *B. belcheri *(Gray). As can be seen in Figure 4c, amphioxus, in comparison to tunicates, shares additional miRNAs with zebrafish and abandons most ancestral miRNAs. These data strongly suggest that amphioxus miRNAs are less divergent from vertebrate miRNAs than are tunicate miRNAs. In agreement with this, the cephalochordate body plan is more vertebrate-like than that of any tunicate, as amphioxus possesses many homologs of vertebrate organs (for example, the pineal and pronephric kidneys) that are not found in tunicates [25]. Thus, the most appropriate organisms to use as a simple model for deciphering the fundamentals of vertebrate development are turning out to be the amphioxus cephalochordates, whose body plans and miRNA repertoires are more vertebrate-like than those of the tunicates. In contrast, tunicates are morphologically and molecularly derived with a trend towards genomic simplification.

## Discussion

One important question in evolutionary biology concerns the origin of vertebrates from invertebrates. Amphioxus is generally accepted as an ideal model to use as a proxy for the ancestral vertebrates [7,8,26]. Recent advances in molecular biology and microanatomy have supported homology of body parts between vertebrates and amphioxus [8,27,28]. Thus, a thorough knowledge of the morphology and genetic programs of amphioxus may provide us with a unique opportunity to reconstruct the major events of early vertebrate evolution and decipher how the vertebrate body plan evolved.

While amphioxus is an outstanding model organism to bridge the huge gap between invertebrates and vertebrates, no amphioxus miRNAs have been registered in the miRNA database miRBase 12.0 [18]. The study of miRNAs in vertebrates such as mice, rats and humans as well as invertebrates such as *C. elegans *and *Drosophila melanogaster *has far outpaced that in amphioxus. Given the important position of amphioxus in metazoan phylogeny, the identification of novel miRNAs from amphioxus will contribute greatly to our understanding of both miRNA evolution and the possible role of miRNAs in facilitating the evolution of more complex animal forms.

Previously, miRNAs were defined as non-coding RNAs that fulfill a combination of expression and biogenesis criteria [10,11]. First, a mature miRNA should be expressed as a distinct transcript of approximately 22 nucleotides that is detectable by Northern blot analysis or other experimental means such as cloning from size-fractionated small RNA libraries. Second, a mature miRNA should originate from a precursor with a characteristic secondary structure, such as a hairpin or fold-back, that does not contain large internal loops or bulges. The mature miRNA should occupy the stem part of the hairpin. By this method, a large portion of the small RNAs, such as breakdown products of mRNA transcripts, other endogenous non-coding RNAs (for example, tRNAs, rRNAs and natural antisense small interfering RNAs), as well as exogenous small interfering RNAs, are filtered out from the population of miRNAs [10,11]. However, hairpin structures are common in eukaryotic genomes and are not a unique feature of miRNAs. Many random inverted repeats (termed pseudo-hairpins) can also fold into dysfunctional hairpins [14,17]. To eliminate the false positive pseudo-hairpins, an optional but commonly used criterion that requires miRNA sequence and hairpin structure be conserved in different species [10,11] was employed in the present study. By this definition, we detected 55 conserved miRNA genes in the amphioxus *B. belcheri *(Gray) that encode 45 non-redundant mature miRNAs. All of these conserved miRNAs meet the expression and structure criteria required for miRNA annotation, and many have additional supporting evidence such as multiple observations of expression, genomic clustering, and cloning of the star sequences. Unfortunately, the problem has not been solved thoroughly since a large number of non-conserved pre-miRNAs with species-specific expression patterns do exist in eukaryotes [16]. To surmount the technical shortfalls of comparative methods for identifying species-specific and non-conserved pre-miRNAs, several *ab initio *predictive approaches have been extensively developed [14,17]. With these methods, many non-conserved miRNAs have been discovered and experimentally verified in viruses and human [14,17]. Here, we used miPred, an *ab initio *prediction approach for identifying pre-miRNAs without relying on phylogenetic conservation, to remove the irrelevant genomic pool of pseudo-hairpins without sacrificing putative non-conserved pre-miRNAs [14,17]. Among 69 pre-miRNA-like hairpins, 11 were classified as pseudo-pre-miRNAs and 58 as authentic pre-miRNAs. Thus, 58 miRNA genes constitute the final collection of non-conserved miRNA genes in amphioxus, and these encode 53 non-redundant mature miRNAs. Likewise, all of these miRNAs meet the expression and structural criteria required for miRNA annotation, and many have additional supporting evidence, including multiple observations of expression, genomic clustering and cloning of star sequences. However, the set of non-conserved miRNAs was fundamentally different from the set of conserved miRNAs, as the non-conserved miRNAs were represented by only 23,613 tags compared to 246,524 tags for the conserved miRNAs. These results indicate that the non-conserved miRNAs are expressed at substantially lower levels or in limited cell types or circumstances.

While we were writing this manuscript, Luo and Zhang [29] reported the computational prediction of 28 miRNAs in amphioxus using a homology search of *Branchiostoma floridae *v1.0 (an incomplete amphioxus genome). However, prediction of miRNAs without experimental proof is not sufficient, since predicted miRNAs only meet the structural criterion for being authentic miRNAs [10]. Furthermore, the computational approach provides no information on the expression levels of amphioxus miRNAs. After carefully comparing our result with that of Luo and Zhang, we found that the dataset from their study is just a subset of the Solexa dataset (Table S10 in Additional data file 1). In addition to computer-aided algorithms, Sanger-based molecular cloning strategies have been frequently used to identify new miRNAs in metazoans [30,31]. By using this method, Dai *et al*. [32] provided experimental evidence for 33 evolutionarily conserved miRNAs and 35 amphioxus-specific miRNAs in the amphioxus *Branchiostoma japonicum*. However, the Sanger-based molecular cloning approach is highly biased towards abundantly and/or ubiquitously expressed miRNAs [17], making it unsuitable for identifying miRNAs that are expressed at low levels, at very specific stages or in rare cell types. This limitation, however, can be overcome by massively parallel sequencing technologies that significantly increase sequencing depth [11]. Accordingly, we employed Solexa sequencing, a massively parallel sequencing technology, to identify miRNAs from amphioxus. Solexa is a breakthrough sequencing technology characterized by numerous distinct advantages over conventional Sanger-based cloning technologies. In addition to avoiding the bacterial cloning steps inherent in Sanger sequencing, Solexa enables hundreds of thousands of short sequencing reads to be generated in one run, thereby boosting the discovery of many expressed small RNAs and resulting in the identification of more candidate miRNAs.

Consistent with this idea, our result is shown to be superior to that of Dai *et al*.: First, the reads of amphioxus miRNAs identified by Dai *et al*. were fundamentally different from ours. For instance, Dai *et al*. identified 841 sequences (out of 2,217 effective reads) as amphioxus miRNAs, whereas we identified 246,524 sequences (out of 313,313 effective reads) as amphioxus miRNAs. Second, after carefully comparing our dataset with that from Dai *et al*.'s study, we found that all the conserved miRNAs identified by Dai *et al*. are just a subset of the conserved miRNAs identified by us, and 23 out of 35 amphioxus-specific miRNAs have been identified by both (Table S10 in Additional data file 1). Third, besides expression and structural criteria, Dai *et al*. provided no additional evidence supporting the correct annotation of amphioxus-specific miRNAs. As can be seen in Table S10 in Additional data file 1, most of the 12 amphioxus-specific miRNAs identified from *B. japonicum *but not found in *B. belcheri *(Gray) are classified as pseudo-pre-miRNAs and represented by a single read. Thus, these non-conserved miRNAs require more careful examination for correct annotation as genuine miRNAs. Fourth, we showed that Solexa can produce highly accurate and definitive readouts of many low-level miRNAs, such as miRNA*s. In contrast, none of miRNA*s has been found from *B. japonicum *by Sanger-based cloning approach. This result further suggests that the Sanger-based molecular cloning approach is unsuitable for identifying miRNAs that are expressed at low levels.

When this manuscript was submitted, miRBase 13.0 was released. Since our analysis was based on miRBase 12.0, we updated the analysis by comparing our dataset with miRBase 13.0. No new miRNAs were identified and none of the major conclusions changed, except that some amphioxus-specific miRNAs were designated corresponding names (Table S10 in Additional data file 1). Taken together, it turns out that Solexa sequencing technology is the most powerful tool for miRNA discovery. More importantly, comparison of miRNA identified from *B. belcheri *(Gray), *B. floridae*, and *B. japonicum *will confirm the existence of some identical miRNAs in amphioxus and provide important clues to the roles of some special miRNAs.

We also present a comprehensive analysis of the organization of amphioxus miRNA genes. Consistent with the miRNA organization in zebrafish, mouse and humans, many amphioxus miRNAs have multiple copies in the genome and/or are organized in clusters. The implications for miRNA gene amplification are still unknown, but miRNA genes with multiple copies may augment or amplify the physiological functions of individual miRNA genes. Our observations support the hypothesis that duplication events causing the rapid spread of miRNA genes throughout the genome occur profoundly in the lineage leading to vertebrates.

Previous studies have suggested that animals with complex organs have increased their cell type repertoire and morphological complexity over geological time in a manner strikingly similar to the expansion of their miRNAs [4-6]. The availability of more miRNAs in animals with complex organs might be helpful to further modulate the developmental network in complex tissues and organs. Interestingly, we noted that although amphioxus does not possess as many miRNAs as vertebrates, it shares a set of key miRNAs with vertebrates that may have had a huge impact on phenotypic diversity and cell lineage decisions during animal phylogeny. For instance, miR-183, miR-184 and miR-96 dominate the population of expressed miRNAs in sensory organs in vertebrates [33], and these were also detected in amphioxus. Consistent with this, amphioxus possesses a frontal eye (homologous to the vertebrate paired eyes) and a lamellar organ (homologous to vertebrate pineal photoreceptors) [28]. Likewise, in agreement with the presence of gastric endocrine cells in amphioxus that are possibly homologous to the pancreatic islet cells of mammals [34], miR-216, miR-217, miR-7, and miR-375, which are characteristic of pancreatic tissue [35], are well established in amphioxus. Although the detailed spatial expression of these miRNAs remains to be shown, it is intriguing to speculate that a pool of such miRNAs contributed greatly to the evolution of complex vertebrate body plans. Further comparison of the body part homology and miRNA repertoires of amphioxus and vertebrates will allow us to model more precisely what our ancestors were like and, thereby, provide a unique opportunity to decipher how the vertebrate body plan evolved.

Another interesting observation is that none of the miRNAs involved in adaptive immunity (for example, miR-181a, miR-155, and miR-223) could be reliably traced back to amphioxus or previous protostomes [36]. When and how adaptive immunity emerged is an evolutionary mystery. It is generally believed that adaptive immunity emerged suddenly and is only present in jawed vertebrates [37]. We hypothesize that certain key miRNAs, such as miR-181a, miR-155, and miR-223, played a fundamental role in the genesis of the molecular machinery of the adaptive immune system. In this regard, the absence of these miRNAs in invertebrates (including amphioxus) explains why adaptive immunity is restricted to jawed vertebrates. However, to understand better the evolutionary origins of adaptive immune systems, more comparative data from jawless vertebrates (for example, lamprey and hagfish) are clearly needed.

## Conclusions

Our current study introduces an accurate and efficient approach for miRNA discovery and will aid the identification of many miRNAs in other species. More importantly, our study provides the basis for future analysis of miRNA function in amphioxus. Further comparison of the body part homology and miRNA repertoire between amphioxus and vertebrates will allow us to model more precisely what our ancestors were like and offer a unique opportunity to decipher how the vertebrate body plan evolved.

## Materials and methods

### Animal collection and RNA isolation

Adults of the Chinese amphioxus *B. belcheri *(Gray) were collected from Beihai, Guangxi, China and kept alive with seawater and sea alga. For Solexa sequencing, 12 adult animals were pooled together, and total RNA was extracted from pooled samples with Trizol (Invitrogen, Carlsbad, CA, USA) according to the manufacturer's instructions.

### Solexa sequencing

The sequencing procedure was conducted as previously described [12]. Briefly, after PAGE purification of small RNA molecules (under 30 bases) and ligation of a pair of Solexa adaptors to their 5' and 3' ends, the small RNA molecules were amplified using the adaptor primers for 17 cycles and fragments of around 90 bp (small RNA + adaptors) were isolated from agarose gels. The purified DNA was used directly for cluster generation and sequencing analysis using the Illumina Genome Analyzer (Illumina, San Diego, CA, USA) according to the manufacturer's instructions. The image files generated by the sequencer were then processed to produce digital-quality data. After masking of adaptor sequences and removal of contaminated reads, clean reads were processed for computational analysis.

### *In silico *analysis

Solexa reads were aligned against the amphioxus genome (*Branchiostoma floridae *v2.0) [9] using SOAP [15]. Sequences with perfect match or one mismatch were retained for further analysis. To further analyze the RNA secondary structures comprising matched Solexa reads, 100 nucleotides of genomic sequence flanking each side of these sequences were extracted, and the secondary structures were predicted using RNAfold [38] and analyzed by MIREAP [39] under default settings. MIREAP is a computational tool specially designed to identify genuine miRNAs from deeply sequenced small RNA libraries; it fully considers miRNA biogenesis, sequencing depth and structural features to improve the sensitivity and specificity of miRNA identification. Stem-loop hairpins were considered typical only when they fulfilled three criteria: mature miRNAs are present in one arm of the hairpin precursors, which lack large internal loops or bulges; the secondary structures of the hairpins are steady, with the free energy of hybridization lower than -20 kcal/mol; and hairpins are located in intergenic regions or introns. Those genes whose sequences and structures satisfied all of these criteria were considered as candidate miRNA genes. Subsequently, we adopted a computational approach named miRAlign to predict new miRNA genes that are paralogs or orthologs to known miRNAs [13]. Finally, all remaining candidates were subjected to MiPred to filter out pseudo-pre-miRNAs. MiPred is a random forest-based method for classification of genuine pre-miRNAs and pseudo-pre-miRNAs using a hybrid feature (including local contiguous structure-sequence composition, minimum of free energy of the secondary structure and *P*-value of randomization test) [14]. Given a sequence, MiPred decides whether it is a pre-miRNA-like hairpin sequence or not. If the sequence is a pre-miRNA-like hairpin, the random forest-based classifier will predict whether it is a genuine pre-miRNA (minimum of free energy <-20 kcal/mol and *P*-value < 0.05) or a pseudo-pre-miRNA (minimum of free energy >-20 kcal/mol or *P*-value > 0.05).

### Stem-loop quantitative RT-PCR assay

Assays to quantify the mature miRNAs were conducted as previously described [19,20]. Briefly, 1 μg of total RNA was reverse-transcribed to cDNA by using AMV reverse transcriptase (TaKaRa Co., Tokyo, Japan) and looped antisense primers. The mix was incubated at 16°C for 15 minutes, 42°C for 60 minutes, and 85°C for 5 minutes. This allowed for the creation of a library of multiple miRNA cDNAs. Real-time PCR was performed using an Applied Biosystems 7300 Sequence Detection system (Applied Biosystems, Foster City, CA, USA) by standardized protocol. In each assay, 1 μl cDNA (1:50 dilution) was used for amplification. The reactions were incubated in a 96-well optical plate at 95°C for 5 minutes, followed by 40 cycles of 95°C for 15 s and 60°C for 1 minute. All reactions were run in triplicate. After reaction, the threshold cycle (CT) was determined using default threshold settings. The CT is defined as the fractional cycle number at which the fluorescence passes the fixed threshold. To calculate the expression levels of miRNAs, a series of synthetic miRNA oligonucleotides with known concentration were also reverse-transcribed and amplified. The absolute amount of each miRNA was then calculated by referring to the standard curve.

### Microarray experiments

The 795 complementary probes (in triplicate) against miRNAs, corresponding to 537 human, 204 mouse, and 54 rat miRNAs, were designed based on miRBase release 12.0 [18]. RNA labeling, microarray hybridization and array scanning were performed as previously described [21]. Briefly, 25 μg of total RNA was used to isolate the low molecular weight RNA using polyethylene glycol solution precipitation. Subsequently, low molecular weight RNAs were labeled with Cy3 and hybridized with miRNA microarrays (CapitalBio Corp., Beijing, China). Finally, hybridization signals were detected and quantified. Four independent adult amphioxus RNA samples were hybridized with miRNA microarrays separately. Hybridization intensity values from individual amphioxus sample were filtered and global median normalized. We considered candidate miRNAs with a signal above 3,000 and *P *< 0.001 from a Student's test (compared with the blank spotting solution) to be positive.

### Pearson's correlation coefficient

Correlation is a technique for investigating the relationship between two quantitative, continuous variables. Pearson's correlation coefficient *R*, also known as the product-moment coefficient of correlation, is a measure of the strength of the association between the two variables. The first step in studying the relationship between two continuous variables is to draw a scatter plot of the variables to check for linearity. The nearer the scatter of points is to a straight line, the higher the strength of association between the variables. The Pearson's correlation coefficient *R *may take any value from -1 to +1.

## Abbreviations

CT: threshold cycle; miRNA: microRNA; pre-miRNA: miRNA precursor; qRT-PCR: quantitative RT-PCR.

## Authors' contributions

CYZ, JZ and KZ conceived and planned the study. XC, QL, and JW worked collectively to develop the strategies and methods described in this paper. QL, LM and JW performed the Solexa analysis. ZR, CW, and YL performed the computational analysis. XG and XJ generated figures and figure legends. GS, XW and JYC contributed to the final manuscript preparation. CYZ and XC wrote the manuscript. All authors have read and approved the final version.

## Additional data files

The following additional data are available with the online version of this paper: Tables S1 to S10 (Additional data file 1); a figure showing the predicted stem-loop structures of conserved amphioxus miRNAs (Additional data file 2); a figure showing the predicted stem-loop structures of amphioxus-specific miRNAs (Additional data file 3).

## Supplementary Material

Additional data file 1 Table S1: categories of amphioxus small RNAs. Table S2: MIREAP output. Table S3: MIREAP summary, miRAlign output and MiPred output. Table S4: mature sequences of amphioxus miRNAs and their cloning frequencies. Table S5: detailed precursor sequences and exact genome locations of all amphioxus miRNAs. Table S6: plausible amphioxus miRNAs. Table S7: comparison of miRNA expression levels in amphioxus determined by Solexa sequencing and microarray analysis. Table S8: raw data of miRNA microarray. Table S9: evolution history of amphioxus miRNAs. Table S10: comparison of miRNA identified from *B. belcheri *(Gray), *B. floridae*, and *B. japonicum*. Click here for file

Additional data file 2 Predicted stem-loop structures of conserved amphioxus miRNAs. Click here for file

Additional data file 3 Predicted stem-loop structures of amphioxus-specific miRNAs. Click here for file
