# Supplementary figures and images for: Identification and characterization of novel amphioxus microRNAs by Solexa sequencing
Source: Genome Biol. 2009 Jul 17;10(7):R78. doi: 10.1186/gb-2009-10-7-r78 (PMC2728532; doi:10.1186/gb-2009-10-7-r78)

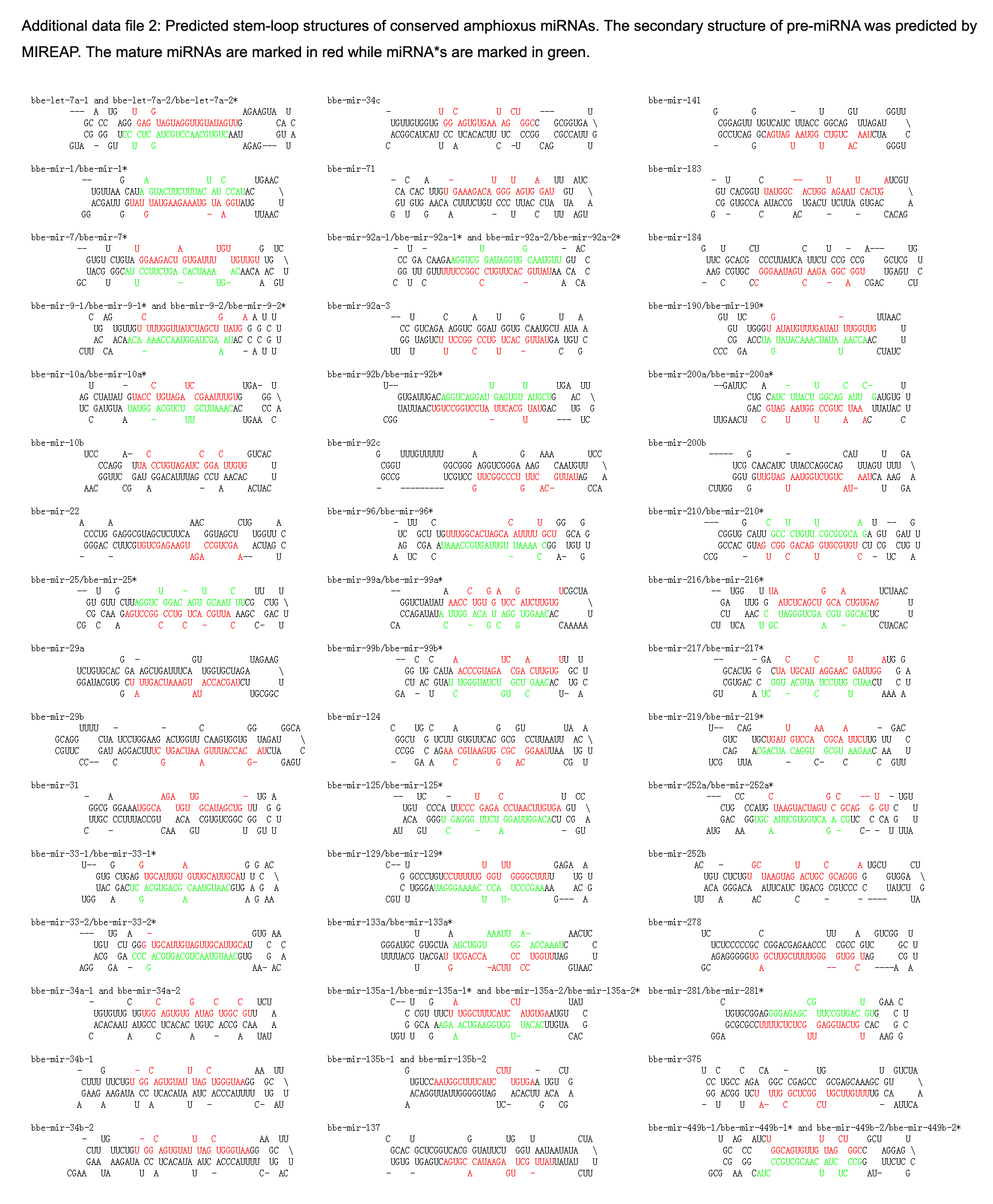

Supplement: Additional data file 2 — Predicted stem-loop structures of conserved amphioxus miRNAs. [file gb-2009-10-7-r78-S2.jpeg]

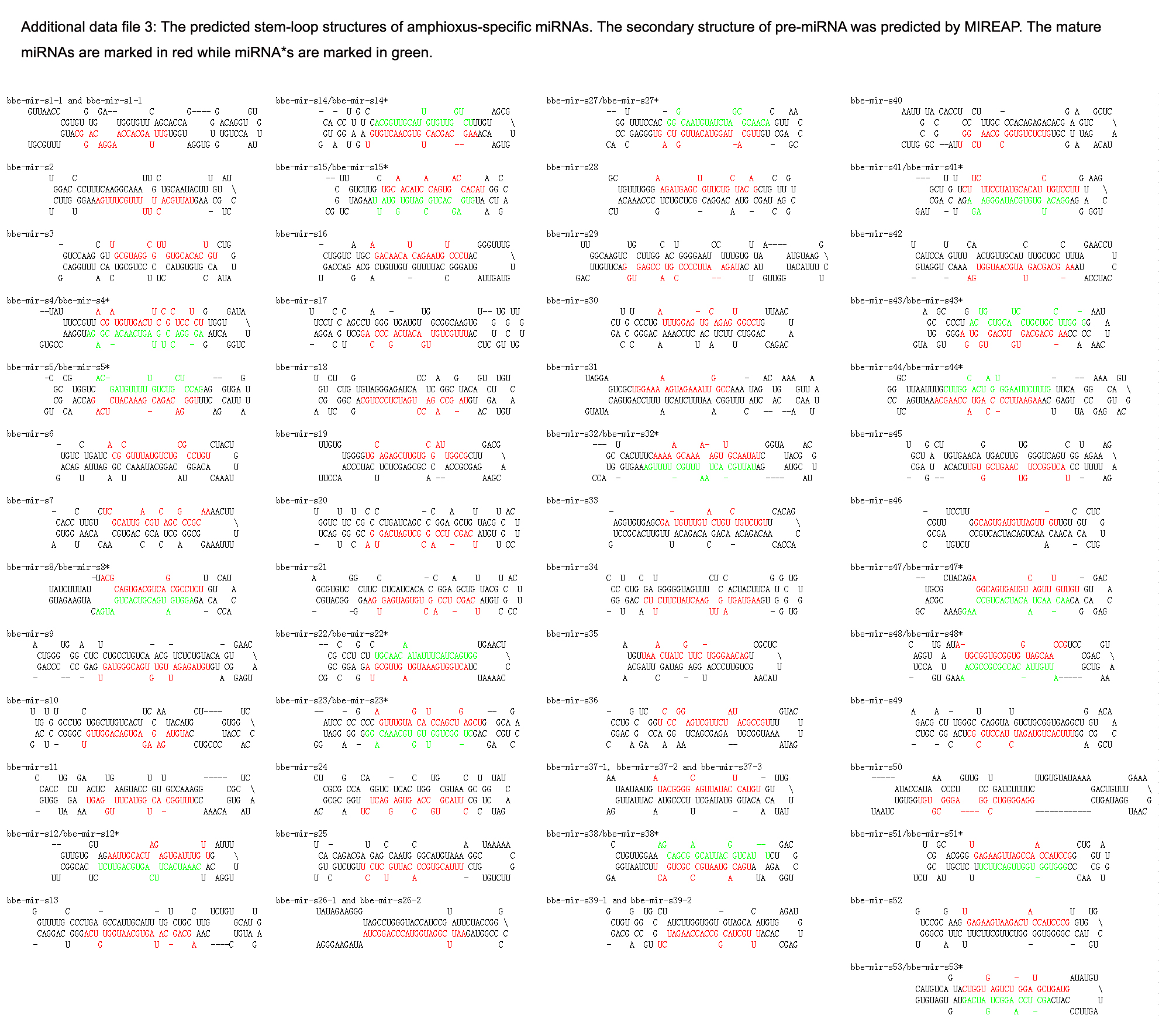

Supplement: Additional data file 3 — Predicted stem-loop structures of amphioxus-specific miRNAs. [file gb-2009-10-7-r78-S3.jpeg]
